# The “Microbiome”: A Protagonist in COVID-19 Era

**DOI:** 10.3390/microorganisms10020296

**Published:** 2022-01-26

**Authors:** Giuseppe Merra, Annunziata Capacci, Giuseppe Cenname, Ernesto Esposito, Maria Dri, Laura Di Renzo, Marco Marchetti

**Affiliations:** 1 Section of Clinical Nutrition and Nutrigenomic, Department of Biomedicine and Prevention, University of Rome Tor Vergata, 00133 Rome, Italy; laura.di.renzo@uniroma2.it (L.D.R.); marco@marcomarchetti.it (M.M.); 2 Department of Medical and Surgical Sciences, Agostino Gemelli General Hospital Foundation-IRCCS, 00168 Rome, Italy; nunziacapacci@gmail.com; 3 Comando Generale Arma Carabinieri, Direzione di Sanità, 00197 Rome, Italy; x@yahoo.it; 4 Department of Human Policies [General Directorate] of Basilicata Region, 85100 Potenza, Italy; x2@yahoo.it; 5 Department of Surgical Sciences, School of Applied Medical-Surgical Sciences, University of Rome Tor Vergata, 00133 Rome, Italy; x3@yahoo.it

## 1. Microbiome, Viral Infections and Frailty

Respiratory infections are among the main causes of hospitalization and mortality, particularly in elderly patients [1]. In this context, the intestinal and respiratory microbiome, whose alteration is related to the concepts of inflammaging and immunosenescence [2], conditions the elderly’s susceptibility to infections. Recent studies show that viral infections, such as influenza and respiratory syncytial virus infection, cause imbalances in the intestinal microbiome (dysbiosis), even in the absence of virions locally. Intestinal dysbiosis is one of the main factors influencing the adaptive response against respiratory pathogens, favoring post-viral pneumonia [3]. Viral infections also act on the respiratory microbiome, causing an increase in the upper respiratory tract of pathogens, such as Staphylococcus aureus and Streptococcus pneumonia [4]. This phenomenon leads to an increased risk of bacterial pneumonia associated with a viral infection. Viral infections, through microbiome metabolites, also facilitate the transition from pathogen to pathogen; an example is the interaction between the flu virus and Streptococcus pneumoniae with the inhibition of biofilm and the stimulation of bacteriokines [5]. It is possible that a deeper understanding of these mechanisms may allow preventing bacterial superinfections during viral epidemics.

The recent COVID-19 pandemic has made the definition of fragility evident and tragically tangible: a condition of increased vulnerability of the individual, determined by multisystemic physiopathological alterations, characterized by a reduced ability to react to stressful situations, with an increased risk of negative events, such as hospitalization and death [6]. Fragility is a multidimensional condition, as clinical, biological, functional and psycho-social factors determine its onset and clinical progress [7]. For this, modernly, the methods of diagnosis and treatment of the frail elderly provide for a multidimensional clinical approach [8]. The difficult management of COVID-19 in the elderly, documented by severe morbidity and by decidedly higher mortality compared to subjects affected by COVID-19 of young or adult age, recognizes multiple factors: the absence of specific therapies; problems related to fragility, such as the presence of multimorbidity and functional and cognitive disabilities; the need for assistance from family members or other caregivers, greatly hampered by the isolation regime; and the risk of adverse events related to polypharmacy. This last factor, above all, complicates the use of experimental therapies (see hydroxychloroquine and immunosuppressants), increases the interest in more “ecological” approaches and is weighed down by fewer side effects, including interventions on the intestinal microbiome.

## 2. Anti-Influenzal Vaccination and Microbiome

Influenza in the elderly is associated with a greater risk of complications and mortality. For this reason, vaccination is promoted, the efficacy of which in the elderly can sometimes be less than in the young person. In fact, a reduced protective efficacy of vaccination could be due, at least in part, to the dysbiosis that is observed in the microbiome during aging and to changes in the adaptive response [9]. A recent meta-analysis reports that the use of probiotics improves the immune response to influenza vaccination, although further studies are needed to clarify the biological mechanism of this phenomenon [10]. A recent study conducted on the cohabitants of patients with viral flu has shown a significant association between the type of respiratory microbiome, particularly some oligotypes, and an increased risk of flu infection [11]. These data suggest an important role of the microbiome in the clinical course of the flu; however, further studies are needed to clarify the pathophysiological mechanisms of these observations.

## 3. Microbiome and COVID-19

Some recent studies suggest a significant association between SARS-CoV-2 infection and the onset of intestinal microbiome dysbiosis, as confirmed by the finding of severe diarrhea (reported in 2–36% of cases) and the presence of SARS-Cov-2 virions in fecal specimens in subjects suffering from COVID-19 [12]. Furthermore, alterations in the intestinal microbiome, characterized by an increase in opportunistic pathogenic germs and depletion of protective commensal bacteria, are associated with fecal levels of SARS-Cov-2 and severity of symptoms from COVID-19 and characteristically persist even after the elimination of SARS-Cov-2 and resolution of disease symptoms [13]. Of great interest is the observation that the severity of the clinical manifestations is associated with advanced age and comorbidity, both elements related to inflammaging and alteration of the qualitative and quantitative composition of the intestinal microbiome. For this reason, it has been hypothesized that an intervention aimed at strengthening the intestinal barrier and reducing the inflammatory stimulus by adopting a diet high in fiber and fermenting foods could be useful in containing gastrointestinal symptoms from COVID-19 [14]. A Chinese study found dysbiosis with a significant reduction in Lactobacillus and Bifidobacterium in patients with COVID-19; however, the clinical significance of this finding is not yet defined.

There is no doubt, however, that alteration of the intestinal microbiome [15] can potentially predispose healthy individuals to an abnormal inflammatory state [16], which can further explain the susceptibility and severity of COVID-19. Scientific evidence suggests that “cytokine storm” may be an important mechanism leading to the severity and death of COVID-19 [17,18] patients. Therefore, anti-cytokine therapy for suppressing patients’ hyperinflammatory status is a recommended strategy for the treatment of patients with severe COVID-19 [19,20]. Increasing evidence has shown that the microbiota plays a fundamental role in the induction, training and function of the host immune system, and the composition of the intestinal microbiota and its activity are involved in the production of inflammatory cytokines [21,22]. Previous studies have reported that the Lactobacillus genus was positively associated with IL-6 and IFN-γ, while the Blautia genus was positively associated [23,24,25].

## 4. Probiotics, Prebiotics and Diet during the COVID-19 Pandemic

Patients with COVID-19 caused by SARS-CoV-2 have various manifestations with severity including enteric involvement. Gut bacteria can help defend against potential pathogens by promoting beneficial immune interactions. A literature review on SARS-CoV-2, COVID-19, gut microbiome and immunity has just been published [26]. In summary, SARS-CoV-2 infection disrupted the integrity of the gut microbiome, and this disruption was associated with the severity of the disease. Subjects with underlying comorbidities who had greater intestinal permeability and reduced gut microbiome diversity had a poorer prognosis. Food microbes, including probiotics or prebiotics, have had antiviral effects against other forms of coronaviruses and could positively impact host immune functions during SARS-CoV-2 infection. Numerous studies are investigating the role of probiotics in preventing and reducing susceptibility to SARS-CoV-2 infection in healthcare professionals, family contacts and affected patients. One approach to strengthening the intestinal barrier and reducing pro-inflammatory states is to adopt a more diverse diet during the COVID-19 pandemic. SARS-CoV-2 infection is associated with immune immunity and changes in the gut microbiota. Although we have the vaccine, probiotics and prebiotics could represent an opportunity for the discovery of microbial therapies to prevent and treat COVID-19.

## 5. Conclusions

As for the data on the use of probiotics in COVID-19, the available results are still too scarce to be able to draw clinical indications. Currently, in fact, the rationale for the use of probiotics in COVID-19 disease derives from indirect evidence. The use of conventional “blind” probiotics does not seem to be recommended until the pathogenesis of SARS-CoV-2 infection and its effects on the intestinal microbiota is understood more fully. However, it is likely that an intervention strategy aimed at modulating the intestinal microbiota may be one of the therapeutic approaches of COVID-19 and its complications [27].

## Data Availability

Not applicable.
